# Bringing Development Back into Development Studies

**DOI:** 10.1111/dech.12490

**Published:** 2019-03-08

**Authors:** Andrew M. Fischer

## Abstract

This article challenges Horner and Hulme's call to move from ‘international development’ to ‘global development’ with a reaffirmation of the classical traditions of development studies. With some adaptation to fit the changing contemporary context, these traditions not only remain relevant but also recover vital insights that have been obscured in the various fashionable re‐imaginings of development. In particular, development thinking and agendas in the past were much more radical and ambitious in addressing the imperatives of redistribution and progressive forms of transformation in the context of stark asymmetries of wealth and power. Such ambition is still needed to address the nature and scale of challenges that continue to face the bulk of countries in the world, particularly given the persistence if not deepening of asymmetries. This reaffirmation is elaborated by addressing three major weaknesses in Horner and Hulme's arguments. First, they do not actually define development, but instead treat it as simply poverty and inequality dynamics, which are better understood as outcomes rather than causes. Second, despite their assertion that the study of (international) development was primarily concerned with between‐country inequalities, this is not true. Domestic inequality was in fact central to both development theory and policy since the origins of the field. Third, the authors ignore the rise of neoliberalism from the late 1970s onwards and the profound crisis that this caused to development outside of East Asia and perhaps India, which the jargon of ‘global’ implicitly obfuscates and even condones. Rather, the experiences of East Asia and in particular China arguably vindicate classical approaches in development studies.

## INTRODUCTION

Following the turn of the new millennium, there was a strong move to rename ‘development studies’ as ‘international development’. This was not without contestation because, among other concerns, the latter term appears too closely associated with international development assistance, reinforcing the common impression that development studies is largely about aid, which of course it is not. Indeed, several development studies departments in the UK were rebranding themselves around that time with titles barely differentiable from the UK government Department for International Development. Nonetheless, this renaming was said to be in the spirit of the millennium times, conveying a more universal sense of interdependence, cooperation and co‐existence among nations. Development studies, by contrast, was perceived to be associated with older colonial or Cold War ways of thinking that some people were anxious to disassociate themselves from. But then what about national and local processes of development? Are they subsumed under what we would call international development? What exactly is the ‘international’ if it is not the relations between nations?

The move towards ‘international development’ in this sense was not well thought out. ‘Development studies’ conveys the sense of something much broader: processes of structural and social transformation that pervade our modern existence and that are fundamental to understand the ways the world has changed over the last two centuries. Understanding these processes and their associated institutional, political, social and cultural manifestations is fundamental to deciphering the evolution of power and wealth at local, national and global scales. It is also fundamental to recognizing the practical prospects for emancipation (rather than just the imagination of emancipation) for those subordinate within those relations, whether under erstwhile conditions of colonization and early decolonization, or else under the more contemporary conditions of what has come to be called globalization. These processes manifest in various ways in various contexts, but a systemic understanding of them remains essential if we are to make sense of this recent experience of humanity, from which we have thankfully not yet extinguished ourselves. The field of development studies, as it was originally conceived, has held a special place in this intellectual endeavour because it takes such modern transformations as the starting point of interdisciplinary inquiry, rather than as an afterthought of core theorization.

Yet now, calls are again being made for a shift from ‘international development’ to ‘global development’, as epitomized by this Forum Debate contribution by Horner and Hulme (this issue). ‘Moving from international to global development’, they argue, ‘is a recognition that we live in “one world” — albeit with major inequalities — and not in a “North” or “South” or in First and Third Worlds’ (p. 368). ‘Such a perspective seeks to account for privilege and prosperity in “developing countries” and for marginalization and relative poverty in “developed countries”’ (p. 368). In other words, you can find the North in the South and the South in the North; both are applicable anywhere, so we might as well call it all global.

Their argument essentially boils down to an assertion that binaries are no longer relevant, largely because of ‘converging divergence’, which refers to decreasing inequalities between countries and increasing inequalities within countries. They frame this as a ‘new geography of development’, drawing heavily on the idea of a ‘new geography of global poverty’. All of this requires a reformulation of development because international development is rooted in notions of binaries and inequality between nations as its ‘historical focus’ (p. 373), and also in international development assistance which is now receding in importance (see, for example, p. 351 or Table 2, p. 372). Horner and Hulme concede that this prior focus cannot be abandoned or ignored given the ‘continued extent of between‐country inequalities’ (p. 373). Nonetheless, they contend that ‘more than at any time over the last century, the contemporary global map of development appears increasingly at odds with any idealized binary notion of a clear spatial demarcation between First and Third Worlds, “developed” and “developing”, or rich and poor, countries’ (p. 349). Hence, their perspective is that ‘development is linked to the whole world. In such a framing, global development is an overarching focus, characterized by a number of dimensions that embrace both international (between‐country) and national (within‐country) issues’ (p. 372). To support their case, they repeatedly note that the Sustainable Development Goals already seem to be making this transition to a more global rather than binary approach to development.

Again, we must question what, exactly, is the ‘global’ in this understanding of global development? Is it about processes that have a global dynamic that cannot be restricted by national boundaries and that should not be conceived in terms of what Gore (1996) once called ‘methodological nationalism’? The most obvious examples are capitalism and demographic transition. But this is no different from classical approaches in development studies, which rooted the study of development within the evolution of capitalism and world order. In any case, this does not seem to be the notion of ‘global’ that Horner and Hulme are implying.

If the case for relabelling is merely based on the idea that relative poverty exists in the ‘North’ and that poorer countries are now the location of substantial wealth, this is also hardly a new phenomenon. The field of development studies was in part born out of the Great Depression of the 1930s, when it was very clear that the leading industrial powers of the world were capable of producing quite broad and intense poverty. Similarly, some of the wealthiest individuals of the 19^th^ century were, in fact, Indian and other national elites in colonized countries, where they were given monopolies and privileges in exchange for their service and loyalty to Empire (see, for example, Washbrook, 1988). That most poor countries today possess a transnational class of around 5 per cent of their population is hardly a ground‐breaking trend, even if the absolute numbers are large (e.g. 5 per cent of China is now 70 million people). The narrative of the new middle classes in poorer countries also needs to be taken with a huge grain of salt given its lack of analytical content regarding the meaning of ‘class’ or even what constitutes ‘middle’ (which Horner and Hulme acknowledge, see pp. 364, 370). However, the fact that development results in rising wealth among increasingly urban populations should hardly be a surprising revelation. It is certainly not one that challenges received notions of development.

Finally, if the idea is that we are now emerging into a more multipolar world, thereby requiring a change of names to mark the occasion, the logic of emphasizing ‘global’ is again questionable for a variety of reasons. Other contributors to this debate have deconstructed much of the argument around the ‘rising powers’ to demonstrate that this is largely a story about China, so I will not belabour this point here.^1^ Rather, I will contribute three additional points, based on three major weaknesses in the argument of Horner and Hulme.

First, within their discussion of nomenclature, Horner and Hulme completely evade the question of what development is. Instead, they simply reduce development to an understanding of poverty and inequality dynamics. This is essentially confusing a description of stylized outcomes with an analysis of causal processes.

Second, it was never the case that the study of development, in its classic sense, was primarily about between‐country inequality. Such inequality obviously described the context in which poorer countries lagged and were subordinated, but development in its origins was primarily understood as a national project of structural and social transformation aimed at overcoming this lagging and subordination, or as ‘catching up’, as recently re‐invoked by Nayyar (2013). Within this project, the issue of within‐country (i.e. domestic) inequality was central.

Third and more proximately, the article ignores the elephant in the room, which is the rise of what is generally referred to as neoliberalism from the late 1970s onwards and the profound crisis that this caused to development outside of East Asia and perhaps India. Moreover, the way that data are marshalled in the article implicitly condones the neoliberal period while hiding the fact that we have been here before and the contemporary resurgence is as much a recovery as it is an advance. And while the exceptional rise of China drives most of the evidence for the convergence thesis of Horner and Hulme, it also vindicates much of the classics of development studies.

## BRINGING DEVELOPMENT BACK IN

On the first point, to their credit, Horner and Hulme do not reduce their understanding of development to the ‘doing’ or ‘making’ of development by ‘practitioners’, as is common in much contemporary scholarship (referred to as ‘big D’ development by Hart, 2009). The problem, however, is that they do not present an understanding of development at all. Rather, they imply development by way of poverty and inequality outcomes. Poverty and inequality dynamics might well be outcomes of development processes, but they do not explain how or why development happens. They might also be the result of something other than development. In contrast, the classic understanding of development in early development studies in the 1940s and 1950s was grounded in an understanding of social and structural transformation. Given that societies around the world have not ceased to transform, this understanding has arguably never lost relevance.

At the risk of appearing pedantic, it is necessary to clarify this foundational understanding given the degree of confusion that reigns in the literature and for which Horner and Hulme offer no clarity. Development, in this classic social scientific sense, refers to long‐run social, political, economic and technological structural transformations that are distinctly modern, although not necessarily conforming to modernization theory. The latter point is important to emphasize because of the facile tendency in the contemporary literature to deride any notion of transition or transformation as guilty of modernization theory or Eurocentric teleology, particularly among more ‘critical’ scholarship (see, for example, Li, 2017).^2^ There is also a recent proclivity to elevate scholars such as Walter Rostow to an intellectual importance that they never possessed (versus an ideological and political importance, which Rostow definitely did have within the US government). Indeed, Horner and Hulme commit this simplification by suggesting that the changing pattern of inequality ‘leaves untenable any notion of development being simply about developing countries (the poor South) emulating the paths previously taken by developed countries (rich North)’ (p. 369). It was only ever the most rudimentary forms of modernization theory (or their reflections in various policy documents or reports by governments or international organizations) that propagated such notions and they were certainly not the generalized basis of developmentalist thinking.

Rather, the seminal scholars of early development economics were by and large very critical of modernization theory and the idea of stages through which all countries travel. Most of these early scholars recognized that, whilst the whole world was going through shared processes of transformation, the challenges of post‐war development were more accurately conceived in terms of lagging and subordination within those transformations. Both lagging and subordination necessarily implied different paths in accordance with fundamentally different structures and positionalities within these transformations. Notably, many of them, such as Arthur Lewis, were also staunch anti‐imperialists and drew from an internationalist standpoint that was increasingly wary of US geopolitical machinations.

Horner and Hulme also cite me (Fischer, 2015) in making their case for global development, with reference to the contemporary relevance of the notion of peripherality. They seem to imply that this justifies a change of emphasis towards greater differentiation at smaller spatial scales and on new categorizations. However, my point in that article (ibid.) was more precisely that the notion of peripherality provides a logic for conceiving of the world according to a North/South binary, or whatever terms one wishes, according to key structural dimensions that are vital to understand wealth and power in the global economy and geopolitics. In this sense, I argued that the ‘challenge of contemporary development studies is to understand this transmutation of lagging and subordination, rather than prematurely succumbing to euphoric narratives of paradigms undone’ (ibid.: 704). It was an argument for the persisting relevance of past frameworks rather than a call for new ways of understanding development.

Within these frameworks, development definitely does imply a fundamental transformation from a pre‐transitional condition to some form of ‘modernity’, whether beneficial, benign or perverse, without necessarily implying that there is a predestined teleological path or outcome within this transformation or that later ‘developers’ will necessarily follow the same path as those that preceded them. Nonetheless, it does imply some discernible structural features of transition.

The most essential are arguably demographic, technological and economic. For instance, Dyson (2001) argues that integral core features of development are found in mortality, fertility and related demographic and sociological transitions, including urbanization. In this regard, the points raised by Horner and Hulme on human development — that crude indicators of health and education have been converging over the post‐war period — have been well‐established in demography for decades. The important observation is that improvements in health in particular have been achieved even in contexts of stagnant incomes and persistent poverty, and hence have been taking place throughout the world to a considerable extent independent of economic performance or even levels of income. As argued by Dyson (ibid.), this implies that the ‘spark’ of demographic transition has been related to technological or other changes that allow for increased control over mortality. Given that these are affordable and easily accessible, they have been disseminating throughout the world at various speeds and are affecting all societies, rich and poor alike, as part of a global process.

On the other hand, technological dissemination in the economic realm is key to the structuring of centres and peripheries within the global expansion of capitalism, in particular because technological capability at the frontier is neither affordable nor accessible. Technological dissemination in this sense does not necessarily determine the growth experiences of countries, even though it operates as a fundamental influence depending on whether a country leads and emanates or follows and receives the dissemination. The two are interdependent, but not necessarily in a deterministic causal manner (determinism meaning that technological dissemination will stimulate capitalist growth in the manner that was experienced by the currently advanced industrial economies). This offers a useful perspective to the dilemma faced by many development theorists, as it is often assumed that ‘modernization’ in one form or another will ultimately lead to fairly deterministic economic outcomes in the long run and, in the absence of those outcomes, modernization must therefore have failed. In other words, societies modernize in terms of demography or even technological consumption without necessarily making their way up the ladder of economic hierarchy.

As concerns the economic hierarchy, technological and economic aspects of modernity essentially refer to the emergence of what Furtado (1983) called ‘industrial civilization’. This is the structuring of an entire economy, including the consumption patterns of its poorer and more marginalized members, around industrial processes and technologies, even if large parts of the economy are not involved in industrial production per se and/or become progressively marginalized from the wealth generated through industrial processes. In many respects, integration into modern ‘industrial civilization’ happens through consumption, not production. As argued by Furtado, the fundamental difference between earlier and later experiences of industrialization — especially post‐war industrialization in practically all developing countries — is that production led consumption in the former, whereas consumption led production in the latter. All developing countries in the post‐war period essentially accessed industrial consumption through imports as the starting point of whatever development path they subsequently followed. With a few exceptions, they continue to do so today with respect to industrial consumption at the technological frontier. It is in this essential sense that they are still peripheries, within this particular, but particularly important dimension.

Accordingly, (capitalist) economic development can be understood as an increasing amount of value‐added produced per person, achieved through increasing labour productivity (output per unit of labour time rather than simply people working more or more people working) and sustained by capital accumulation, both at a relatively broad economic scale. At an aggregate level, increasing labour productivity usually results from synergies of increasing degrees of specialization and scales of production, as well as increasing applications of technology in production processes (including social organization, which can be seen as a social form of technology).

The precise combinations of these elements at various scales vary depending on patterns of integration into regional, national and global economies. Indeed, some subnational regional economies might possess few or none of the attributes of increasing productivity in agriculture or manufacturing, but nonetheless experience the effects of a rise in national labour productivity, in particular through the supply of cheap mass‐produced goods, the circulation of monetary aggregate demand, migration, and the dissemination of wage norms throughout the national economy supported by government fiscal policy. Indeed, aid transfers can also be understood as another way to raise incomes and hence modern consumption without necessarily entailing economic development.

However, it is also difficult to know where to draw the regional boundaries of economic development, that is, the boundaries within which a region can be considered to be developing economically on the basis of local, regional, national or global capitalist processes. Notably, there are usually wide swathes of territory and large proportions of population even in advanced industrial countries that are based mostly on the consumption of modern goods rather than their production (increasingly so as employment continues to shift into services). Similarly, many poor countries, such as in sub‐Saharan Africa, experience transformation through consumption‐driven integrations into ‘industrial civilization’ without necessarily being involved in industrial modes of production, besides in enclave sectors such as mining. Such patterns of integration can lead to many paradoxical dynamics within the labour transitions of such peripheral economies, that is, paradoxical from the perspective of our understanding of labour transitions from a classical European perspective.

In this sense, it is important to break out of simplistic associations regarding development. It is not as if negative attributes (such as persistent poverty) or even a lack of structural transformation in some sectors or subregions of an economic system (such as the persistence of low productivity economic activities in rural areas) somehow invalidate the existence or possibility of development. Rather, from a broader perspective, it is important to consider how such attributes are the dialectical parts of a developmental whole, especially with regard to the integration of peripheral regions within wider economic systems. In particular, the ways that development manifests in peripheral settings can be quite different from the ways it manifests in more central locations, although this cannot be understood in abstraction of how the system as a whole is structured and continues to transform over time.

As Raùl Prebisch outlined in his centre–periphery approach (UN, 1950), peripheries tend to exhibit certain common features of economic structure, conditioned by the propagation of technical progress that establishes the outward‐directed, externally propelled development of peripheries. Disarticulated, fragmented and specialized production structures tend to produce a wide and heterogeneous range of productivities, which provides the structural foundation for higher inequality given the large spread of wages and productivity. It was these features that led Prebisch to suggest that two of the four characteristics of peripheral capitalist economies are marginalization of disadvantaged populations in the peripheries and imitative metropolitan consumption patterns of periphery elites.

The contemporary relevance of these foundational ideas can be seen, for instance, in current debates on the enclave‐nature of foreign direct investment in mining in Africa. Some authors, such as Kaplinsky et al. (2011), dispute the enclave characterization of this sector insofar as the outsourcing of most stages of the value chain to independent firms has replaced traditional vertical integration within the sector.^3^ However, this somewhat misses the larger point of the classic structuralist critique, in that enclaves are but one manifestation of the tendency for polarization within peripheral economies. In other words, outsourcing within a particular sector does not necessarily address the heterogeneity of economic structures at wider economic scales, which the manner of outsourcing might in fact reinforce.

These foundational approaches to understanding development are still fit for purpose. The conditions that have allowed for the generalized resurgence of growth in the last decade or so have been based on a particular conjuncture of commodity booms and intense financialization, which have counteracted the austerity and punitive pro‐cyclical disciplining faced by most developing countries during the neoliberal heydays of the 1980s and 1990s. However, that this would tend to exacerbate polarization within the domestic sphere of such peripheral economies has been a long‐established insight.

## ON THE CENTRALITY OF DOMESTIC INEQUALITY THAT ALWAYS WAS

On the second point of critique, it should be obvious from the above that it was never the case that the study of development, in its classic sense, was primarily about inequality between countries. Such inequality described the lagging and subordination of poorer countries, but development studies in its origins was focused more precisely on solving the problem. This was necessarily a domestic affair, understood as a national project of structural and social transformation, relying mostly on domestic resources, and informed by anti‐imperialism and a commitment to self‐determination. At a collective scale, this could only ever be achieved by a state.

Within this project, domestic (within‐country) inequality was a central consideration. The idea that somehow it was not, has possibly been induced by gross misrepresentations of Simon Kuznets and Arthur Lewis, among others. For instance, Milanovic (2011: 9), one of the current doyens in the study of international inequality, claims that Kuznets argued that ‘in preindustrial societies, almost everybody is equally poor so inequality is low’. Piketty (2014) also misrepresents Kuznets in similar ways.

Kuznets actually argued the opposite. In his seminal article, Kuznets (1955: 25–26) doubted the relevance of his hypothesis for ‘underdeveloped’ countries on the basis of the fact that they generally had higher levels of inequality, not lower, and at lower levels of income than was previously the case for the richer countries he was studying.^4^ He suggested that the impact of an increase in inequality is far sharper because most of the population is closer to the absolute threshold and the concentration of savings is higher. These features were perpetuated by an absence of dynamic forces that checked the upward trend of upper‐income shares and of political and social systems to increase the lower‐income shares in such developing countries (i.e. fiscal and welfare systems were very weak in most cases). He also presciently noted that financial liberalization would exacerbate these conditions by facilitating the flight of elite wealth abroad or into real estate. These observations led Kuznets to seriously question the presumptions of modernization theory, contrary to how he is often portrayed.

The idea that domestic inequality is somehow newly relevant has also been influenced by the narrative of new geographies of global poverty, on which Horner and Hulme rely so heavily. This recent idea that the geography of poverty has changed and that the majority of the world's poor people now live in middle‐income countries was first innovatively unearthed and explored by Sumner (2010), who has been hugely successful in entering this idea into the upper echelons of policy consciousness and discourse. The idea has now reached the status of an accepted stylized fact.

It must be noted, of course, that this is really about a threshold effect rather than geography. In other words, a number of large countries passed from lower‐income country status to lower middle‐income country status (according to the World Bank Atlas method of calculating per capita gross national income). For instance, India passed from US$1,000 in 2000 to US$1,110 in 2009, thereby passing the threshold of lower middle‐income country status (which was set at US$1,026 as of 2017). Indeed, Sumner discusses this in his work and also stopped referring to geography after his first several papers, but this has been largely ignored in the popular uptake of the idea.

Sumner (2016) nonetheless suggests that this change in status fundamentally changes the distributive logic facing such countries, in terms of the amount of domestic resources (per capita) that are now available to address poverty in these countries. This contrasts with lower‐income countries that lack such domestic resources and therefore must rely on aid (or at least, that more readily lend credibility to the case for aid). This is supported by Hoy and Sumner (2016) who show that most countries actually have the resources to eliminate a large part of extreme poverty.

While the threshold is arguably not that significant in terms of fundamentally altering the distributive and redistributive options facing these countries, the more important point is that the essence of post‐war development debates was *always* about national distribution and redistribution. National liberation leaders and movements were intensely aware of the need to redistribute highly concentrated wealth inherited from the colonial era in support of decolonization, which was encouraged by the socialist proclivities of many of these movements. Redistribution was also one of the main messages of the Latin American structuralists in the 1950s, as a crucial means to enhance domestic demand and integration, especially as they became more and more critical of import substitution industrialization policies that were being usurped by strategic doses of transnational corporate investment and ownership. Redistribution was also the big lesson from South Korea and Taiwan, where extensive state‐led land reform was implemented at the beginning of their post‐war development trajectories, while they were still very poor, not once they had attained middle‐income country status (see, for example, Kay, 2002; Putzel, 2000). The reasons why some African countries that attempted similar strategies (e.g. Tanzania) apparently failed remain contested in interpretation (see, for example, Gray, 2018), although the principle of strong asset redistribution remains a powerful lesson from the few obvious cases of post‐war development success.

One vital reason for this is because the domestic resource mobilization strategies that were common among poor countries in that era, such as policies to extract surplus from rural populations in order to subsidize and finance industrialization, were possible only in the absence of high levels of rural inequality. Otherwise, in the context of high inequality, as was typical in Latin America, policies of rural surplus extraction would tend to drive the rural poor into even further crippling poverty. Such outcomes were evidenced, for instance, by the way that colonial strategies of rural surplus extraction caused widespread and intense famines in India (Davis, 2001; Mukherjee, 1974). Indeed, this is similar to the point raised by Kuznets regarding the sharper impacts of rising inequality in very poor and unequal settings.

In any case, such strategies never stood a chance in high inequality countries given that they were blocked by powerful wealthy constituencies of landowners and associated elite factions, which is a political economy characteristic typical of high inequality settings. US‐supported *coup d’états* (e.g. Guatemala 1956, Chile 1973, etc.), and its support to right‐wing military regimes opposing surprisingly successful revolutionary movements (e.g. El Salvador and Guatemala from the 1970s to the 1990s), also made clear that domestic inequality was definitely on everyone's radar then.

This calculus also applies to China. Both collectivization in the Maoist period and de‐collectivization in the immediate post‐Maoist period established a very strong equalization in the use of land assets among the rural population. The development strategies pursued from the Maoist period up to the 1990s, such as the implicit subsidization of urban industrialization by rural areas through a variety of price and financial mechanisms,^5^ arguably could not have been pursued if the starting point in the late 1970s had already been at a high level of inequality, as it had been under the Nationalists (*Guomintang*) prior to their defeat in 1949.

This role of redistribution in setting the stage for domestic resource mobilization strategies has always been relevant for very poor countries; it has not required their emergence into middle‐income status in order to make it so. The focus on thresholds as signifying some sort of fundamental change also distracts us from the vital synergies that can be observed in successful historical cases of development between simultaneous or closely sequenced public interventions in both domestic redistribution and production. These points are generally ignored in the contemporary revival of attention to domestic resource mobilization, such as in the current Financing for Development (FfD)^6^ agenda, particularly as donors retreat from a variety of aid‐dependent countries in Africa. Rather, the main default strategy for domestic resource mobilization is through value‐added taxes, which are generally considered regressive. As such, these current agendas lack ambitious transformative visions or programmes that could render the aim of domestic resource mobilization as socially and politically sustainable or legitimate.

## DEVELOPMENTAL DÉJÀ VU

Following from the above, it is important to recall that we have been here before. The elephants in Horner and Hulme's room are the conditions that led us away from that earlier point. These are the rise of austerity, structural adjustment and neoliberalism^7^ for a large number of developing countries as one particular creditor‐biased way to aggressively overcome the systemic impasse of the 1970s.

The context of the 1970s is important to recall. Rapid even if unstable growth in most developing countries was closing the gap with stagnating rich countries. There were strong calls and serious organization around the idea of a New International Economic Order. As noted above, demands for domestic redistribution in developing countries were also becoming increasingly prominent in the 1970s, in particular from relatively successful revolutionary movements (some socialist, others not) and from serious challenges to US hegemony and to US support for right‐wing regimes around the world, such as in Vietnam, Iran and Nicaragua. International organizations were also shifting towards greater advocacy for redistribution, coined as development with equity or development with a human face. The influence of the World Bank and the International Monetary Fund was quickly being eclipsed given their impotency in being able to regulate the anarchic international monetary system that emerged after the US went off the gold standard in 1971, and because unprecedented supplies of affordable dollar liquidity were freeing middle‐income countries from the need to rely on official finance. Much like today, many people were predicting the eventual redundancy of these Bretton Woods institutions, or else calling for serious reforms to make them more representative of the emerging new order.

The 1982 debt crisis then marked the decisive turning point of US global hegemonic revival, at least with respect to the rebellious ‘Third World’, ending its slow erosion of control on economic, political and military fronts during the turbulent 1970s. It ushered in the ‘changing of the guard’ in the World Bank, replacing the earlier (American) structuralist influence of Hollis Chenery with the neoliberal ascendance of Anne Krueger. The tide also turned within the field of development studies, although not without much contention and resistance, if only because the focus was forcibly shifted toward economic crisis and depression, which UNICEF coined as ‘adjustment with a human face’ (Cornia et al., 1987). These shifts reflected the demise of developmentalism in most low‐ and middle‐income countries outside South and East Asia, and of the Third Worldist activism and confidence that had been effervescing throughout the 1960s and 1970s. Most of Latin America, Africa and West Asia had been effectively disciplined through international finance in a way that could not be achieved through direct politics or military intervention, particularly following the US debacle in its war with Vietnam.

However, it is important to recall that radically different paths could have been taken. The idea that ‘there is no alternative’ (TINA) to the one taken is the ideological construct of this US‐led reassertion of hegemony. Indeed, one of the more problematic aspects of the lexicon of globalization that emerged in the 1990s is that it feeds into this TINA doctrine. The term is generally used to refer to the post‐1980 period and, in particular, the post‐Cold War neoliberal euphoria in the 1990s. As such, it effectively serves as a depoliticizing device in the discussion of policy, similar to the role of development goals, as argued in Fischer (2013, 2018b). In particular, it makes the neoliberal path appear as an inherent and natural aspect of globalization, or as determined by globalization. If one accepts the good aspects of globalization, such as increased human mobility, improved global communications and the dissemination of medical technologies, it must necessarily follow that one accepts other aspects that effectively equate to a neoliberal ideological and policy position, such as privatization, deregulation, and trade, financial and capital account liberalization. It is in this sense that the anti‐globalization movement arguably succumbed to forfeiting the narrative battle given that what has really been at stake is the nature of policy, not whether globalization is inherently a good or bad thing. The ‘global’ rhetoric in this sense distracts us from fundamentally challenging or even addressing the neoliberal paradigm, let alone capitalism.

As is common with many of the well‐known millennial books on poverty, the economic data presented by Horner and Hulme are also predisposed towards a revisionist reading of how the world has changed in the post‐war period, in particular by obfuscating the development gains and then losses of the past. With one exception (Figure 1, p. 350), the economic data start in the late 1980s or 1990s, in line with standard World Bank practice and undoubtedly because the authors rely almost entirely on World Bank data. In so doing, they hide the important development gains that were made up to the 1970s and the damage that was done to development in the 1980s. They also distort the perception of growth since the early 2000s, much of which more accurately represents recovery once the very austere and punitive international conditions facing many countries were relaxed.

For Latin America and sub‐Saharan Africa in particular, the two world regions that were most subjected to crisis, austerity and structural adjustment in the 1980s and 1990s, a start year of around 1990, as was institutionalized by the Millennium Development Goals, represents about the worst point in a disastrous and highly contentious period. Poverty rates notably started to rise in these two regions from the late 1970s onwards and, in Latin America, reached their apex around 1990.^8^ From this trough of depression, things could have presumably only gotten better (although they generally did not for a while more). A large proportion of poverty reduction since this time (about half in the case of Latin America) effectively represents the recuperation of lost ground from previous development gains, following the debilitating assaults during the 1980s on the means that were used to achieve these previous gains.

The choice of start dates around 1990 in this sense lends itself to interpreting any recovery from deep economic depression as a vindication of the prevailing international economic and political system that contributed to the depression in the first place. This includes the role of leading international financial institutions (IFIs) in engineering the severe austerities imposed on the debtor countries. There was some internal dissension in the ranks of the IFIs when times were still dire up until the early 2000s, but these were mostly admissions that they had been too fervent, not that the direction taken was wrong (for a recent example, see Ostry et al., 2016). As noted by Mkandawire (2014) in the case of Africa, the subsequent revival of growth also revived self‐celebratory narratives that structural adjustment nonetheless provided the foundation for growth recoveries through the imposition of sensible discipline and constraint on national governments (in contrast to the simultaneous deliria occurring in international finance, which seemingly faces no discipline or constraint). The possibility that growth recovered simply as a result of relaxing the shackles, allowing countries to start breathing financially again and to return to their interrupted development endeavours, is rarely considered within such self‐congratulatory narratives.

In contrast, the sheer lack of analysis of the pre‐1980 period is notable in these exercises of policy‐based narrative creation. This is again probably due to the predominant practice of relying on the data of the World Bank International Comparison Programme (as do Horner and Hulme), which only starts around 1980. Indeed, the World Bank purchasing power parity (PPP) poverty rates would lead us to believe that almost the entirety of China's population (i.e. over 80 per cent) was on the verge of starvation in 1981. Even though the country was very austere at the time, it is highly unlikely that more than eight out of ten people were living in a state that is best described as one of hunger or starvation. Instead, as discussed in Fischer (2018b), it is more likely that these poverty measures (and their more recent revisions) are very poorly adapted to reflect the profound structural and institutional transformations occurring since the early 1980s in China. Through these transformations, the nature and experience of poverty has transformed in profoundly, in particular with respect to the commodification of health care, education or housing, as well as the breakdown of various provisioning systems that, despite the evident austerity of the late‐Maoist economy, would have nonetheless permitted a decent level of health and other basic needs. In this manner, the linear transposition of current metrics into the past obfuscates the important development improvements that were key preconditions to China's emergence in the 1980s, particularly in areas of human development, such as the huge improvements in health and education in the 1960s and 1970s. Instead, the linear transposition reinforces narratives that China was essentially a basket case until it liberalized and privatized.

These points need to be stressed because the story about China, as well as the story about recent resurgent growth in the South more generally, is much better read as a vindication of many of the classics of ‘old’ development economics — before it was replaced by what Hirschman (1981) famously referred to as ‘mono‐economics’ — rather than as a cause for putting into question their relevance. The exercise of re‐fashioning the semantics distracts us — in particular students and young scholars — from the important lessons we can draw on, thereby doing a disservice to the legacy of our field by encouraging an amnesia which is happily exploited by others, whether neoliberal or post‐developmental.

This amnesia is particularly unhelpful in the current conjuncture when those countries that are struggling to attain middle‐income status and/or remain there are again potentially facing bouts of crisis similar to those that brought down the previous era of developmentalism. As was classically postulated by early development economists such as Prebisch on the basis of practical experience, a fundamental dilemma facing development strategies is that they tend to exacerbate external vulnerability. This is not necessarily because of bad policy or behaviour, but because of structural economic dependence, whereby the intensification of development strategies tends to intensify reliance on foreign exchange (see Fischer, 2018a for an elaboration of this point). Hence, it is no coincidence that ‘Rising Africa’ has come with sharply rising African external debt or foreigners holding domestic debt (see, for example, Akyüz, 2017), much of it with interest rates that would cause economic recession or worse in the US or Europe. As in the past, the alternative of relying on foreign direct investment has also come at the cost of a rapid deepening of dependency in economic structures. That both would come with a rise of domestic inequality is not particularly surprising.

## CONCLUSION

Any exercise of labelling development should logically begin with trying to understand what development is, and not with a description of its outcomes (or of the outcomes of other things such as crises for that matter). Horner and Hulme avoid this task. They simply reduce their understanding of ‘international development’ (as if this label had already become established convention) to North–South binaries and between‐country inequality. They then propose ‘global development’ as an appreciation of a nuance that was, in fact, always there, and a rejection of binaries that are, in certain important respects, still with us.

Relabelling in this sense is problematic because it sets up a false caricature of past thought. In the process, it does a disservice to the field, in particular because so many of the classical concerns and lessons from the tradition of development studies remain so relevant for understanding contemporary problems, for poorer countries and even for those that have attained middle‐income status. Indeed, there remains a huge difference between the disparities and perversities of rich countries, and the real challenges of development that are still paramount to the large majority of countries around the world, regardless of their diversity and regardless of the fact that they contain some rich people.

Development studies — as it was and could continue to be — retains value as an interdisciplinary field because it starts from an understanding of transformation as the foundation of theorization and analysis, and how associated social, political, economic and cultural dynamics interact with and are conditioned by such transformation. Other social science disciplines or interdisciplinary fields could supplant this role, but so far they have not. It is time to reaffirm the field in this purpose.

This partly involves recognizing that development was always conceived primarily in terms of national or collective projects of structural and social transformation in contexts of lagging and subordination within globalizing capitalism. It is relevant here to use the term ‘global’ with respect to capitalism because it is the operations of large transnational corporations and international finance that have truly become global, albeit while still retaining a centre of operations, where profits eventually come home to roost after passing through numerous subsidiaries and affiliates in offshore financial centres. However, this does not diminish the importance of the national collective project, even though it changes the nature of the challenges that such projects now face, particularly with respect to practising industrial policy or maintaining control over fiscal systems in the context of liberalized financial and trade regimes. The goal might be catching up, although merely keeping up with changing structural and institutional norms is already a massive task in most cases.

Within such projects, domestic inequality remains a crucial issue, even for very poor countries. This is partly because it is very difficult to engage in domestic resource mobilization strategies in contexts of high inequality, for reasons of poverty, political economy and legitimacy, and also because of the underlying structural tendency within peripheral economies towards domestic polarization. This point is overlooked in the current FfD agenda and, in the lacuna, the main default strategy for domestic resource mobilization is through value‐added taxes — which are generally considered regressive — in the absence of any ambitious transformative agendas. It would be important to revive the classical attention to inequality and redistribution, but not necessarily for the reasons that Horner and Hulme stipulate, which reproduce an amnesia of the past.

Moreover, through relying heavily on World Bank data and narratives, Horner and Hulme implicitly condone the neoliberal period as one of problematic and yet tentatively emancipatory change. This is characterized as the South gradually and diversely rising up to the North, although at the cost of increasing differentiation within the South, both across countries and within them. Yet, this fundamentally distorts the perception of how the world has changed in the post‐war period, with respect to important development gains that were made up to the 1970s versus the development collapses that occurred in the 1980s and 1990s outside of East and South Asia. And while the narrative of a fundamental shift now is definitely inspired by the impressive return of China to global prominence after a 200‐year eclipse of its geopolitical importance, even this tends to be subverted into the implicit condoning of neoliberalism, or at least serves to obfuscate the neoliberal period for everyone else. It avoids the fact that China's experience more or less vindicates classical approaches to development, whereas crises everywhere else also vindicate these approaches in their abandonment.

## Footnotes

1 For critical reflections on the case of China, see Fischer (2015) or Yu (2013).

2 Indeed, in her three teleological stories of development, Li (2017) sidesteps the fact that Marxism is also teleological, and that a story of crisis can be as teleological as a story of progress within an understanding of capitalism.

3 I am indebted to Ben Radley for pointing out these debates to me.

4 Lewis (1954) similarly noted that inequality in overpopulated underdeveloped countries is usually higher than in advanced industrialized countries because agricultural rents are so high.

5 For a recent discussion of these strategies in the Maoist period, see Eisenman (2018).

6 For instance, see: www.un.org/esa/ffd/; www.worldbank.org/en/about/partners/maximizing-finance-for-development, or http://pubdocs.worldbank.org/en/822151531513670691/FinancingforDevelopmentattheWBGBrochureJuly2018Edition-Updated-July-22-2018-reduce-file-size-final.pdf

7 While the term neoliberalism has admittedly been overused and abused, here it is used in its narrow political economy sense, referring to the ideological creed that policy should strive towards creating or imitating perfectly competitive markets as much as possible. In practical terms, this roughly equates to a combination of monetarist, purportedly neutral but often pro‐cyclical macroeconomic policy, the trio of privatization, liberalization and deregulation, and attempts to introduce market‐type or market‐mimicking mechanisms into spaces that operate according to different logics, particularly in the public sector, such as New Public Management.

8 For Latin America, for example, see Cepalstat.
